# Valsalva Retinopathy in a Young Patient: A Case Report

**DOI:** 10.7759/cureus.83039

**Published:** 2025-04-26

**Authors:** Larissa de Oliveira Silva, Lucas Neves de Oliveira, Mateus Neves de Oliveira, Hermelino Lopes de Oliveira Neto

**Affiliations:** 1 Faculty of Medicine, Universidade Estadual de Feira de Santana, Feira de Santana, BRA; 2 Faculty of Medicine, Escola Bahiana de Medicina e Saúde Pública, Salvador, BRA; 3 Ophthalmology, Universidade Estadual de Feira de Santana, Feira de Santana, BRA

**Keywords:** acute visual loss, pars plana vitrectomy (ppv), premacular hemorrhage, retina hemorrhage, valsalva retinopathy

## Abstract

Valsalva retinopathy (VR) is a rare condition characterized by sudden visual loss due to premacular hemorrhage caused by abrupt increases in intraocular venous pressure during the Valsalva maneuver. It primarily affects healthy young adults during activities such as weightlifting, intense exercises, vomiting, and coughing. Spontaneous hemorrhage resolution may take several months, during which dehemoglobinization occurs gradually through macrophage activity - potentially leading to chronic visual impairment. This case report discusses a 20-year-old male with sudden vision loss in the right eye after weightlifting. Initial visual acuity was counting fingers at 10 cm, with premacular hemorrhage confirmed by ocular ultrasound. Expectant management was initially chosen; however, due to the lack of improvement after 60 days, pars plana vitrectomy (PPV) was performed. Postoperatively, the patient achieved 20/20 visual acuity with no retinal abnormalities. The pathophysiology of VR involves rupture of the perifoveal capillary plexus due to increased intrathoracic pressure. The macular region is prone to hemorrhage due to the lack of firm internal limiting membrane adhesions. While spontaneous resolution is common, large hemorrhages can cause complications such as macular holes, epiretinal membrane formation, and loss of photoreceptors due to the generation of free radicals. Treatment options include laser membranotomy and PPV, with the latter being preferred for dense or persistent hemorrhages. In this case, PPV successfully restored the patient’s visual acuity, underscoring the importance of timely surgical intervention when conservative management fails. Early ophthalmologic evaluation and appropriate clinical follow-up remain critical in managing VR and preserving vision.

## Introduction

Valsalva retinopathy (VR) is an uncommon but well-documented condition caused by a sudden increase in intrathoracic and intra-abdominal pressure. This abrupt surge leads to a transient elevation in intraocular venous pressure, typically triggered by Valsalva maneuvers such as heavy lifting, forceful coughing, vomiting, or straining. The pressure gradient compromises the structural integrity of the perifoveal capillary plexus, resulting in a hemorrhage that often localizes to the sub-internal limiting membrane (ILM) or subhyaloid space [1,2].

These hemorrhages tend to accumulate in the macular area, a region particularly susceptible due to the relatively weak adhesion between the ILM and the underlying retina - an anatomical feature that facilitates blood pooling in this central zone [3,4]. While the initial mechanical rupture is the primary event, secondary biochemical mechanisms may further contribute to visual deterioration. Prolonged contact between the retinal surface and blood components - particularly hemoglobin and iron - can induce the release of reactive oxygen species, promoting oxidative stress and potential damage to the photoreceptors and retinal pigment epithelium [5,6]. Over time, this process may lead to structural complications such as epiretinal membrane formation or, in some cases, macular hole development.

In most patients, particularly those with small or moderate hemorrhages, spontaneous reabsorption occurs without intervention or lasting sequelae. However, in cases involving dense or centrally located bleeding, especially when visual recovery is delayed, treatment may be necessary. Management strategies range from close observation to Nd:YAG laser membranotomy or pars plana vitrectomy (PPV), with the choice guided by the location, density, duration of the hemorrhage, and associated risks [7,8].

Here, we present the case of a healthy young male who experienced sudden visual loss following a weightlifting session. He was found to have a dense premacular hemorrhage consistent with VR. After two months of conservative follow-up with no visual improvement, the patient underwent vitrectomy with complete anatomical and functional recovery. This case illustrates important diagnostic and therapeutic considerations in the management of persistent VR and underscores the value of individualized care in optimizing outcomes.

## Case presentation

A 20-year-old Caucasian male presented to the emergency department complaining of visual blurring in the right eye (RE) for the past three days following an intense weightlifting session. He reported central visual field loss in the affected eye, with preservation of peripheral vision. There was no associated ocular pain, history of trauma, or prior ocular/systemic disease. Best-corrected visual acuity (BCVA) was hand motion in the RE and 20/20 in the left eye (LE). Intraocular pressure measured at 2:00 PM was 10 mmHg in both eyes. Anterior segment biomicroscopy was unremarkable bilaterally.

**Figure 1:**
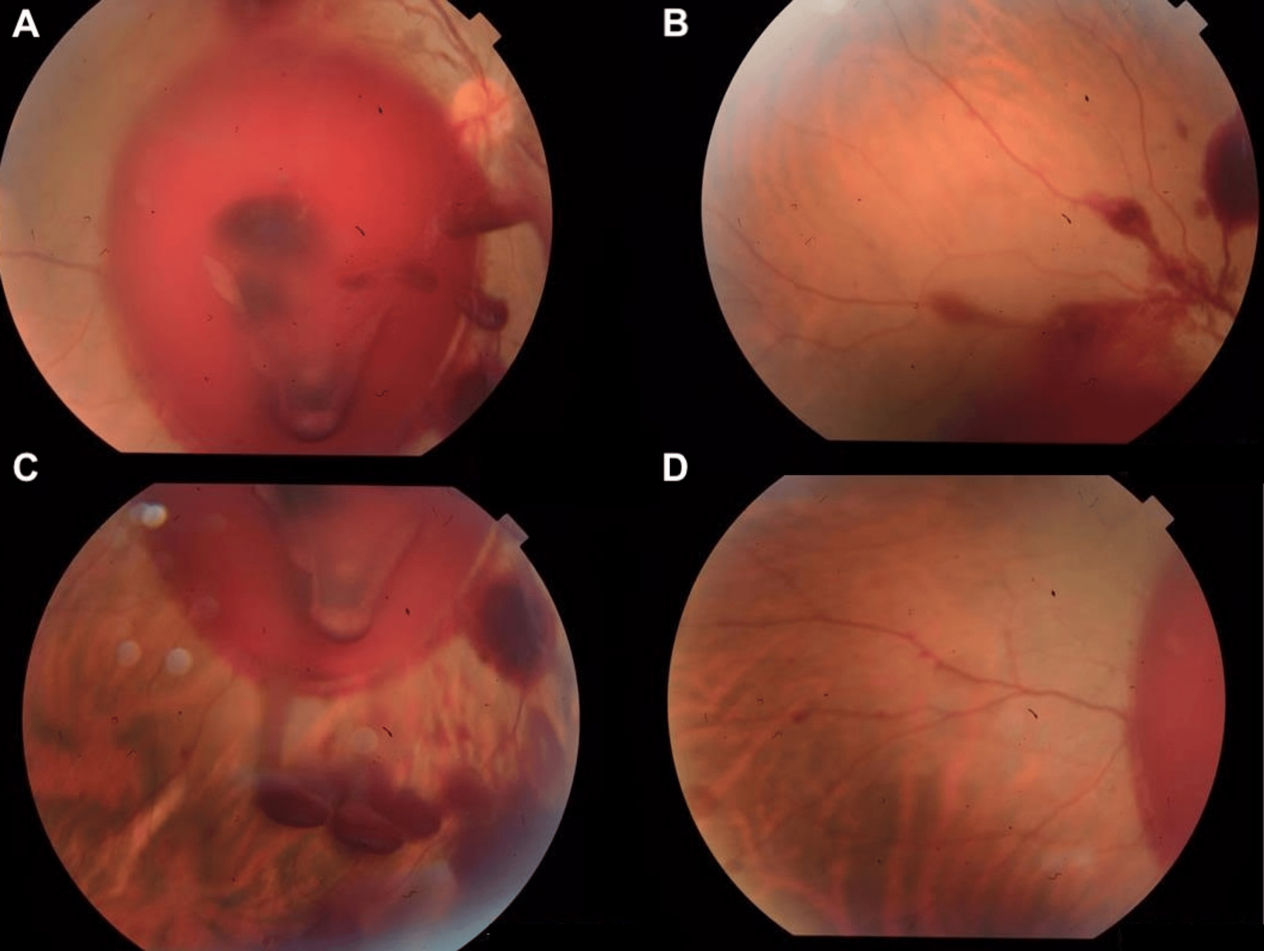
Initial retinography revealing an extensive subhyaloid and vitreous hemorrhage, approximately six disc diameters in size, involving the entire posterior pole (A) and extending toward the peripheral retina (B-D)

Fundoscopic examination of the RE revealed an extensive subhyaloid and vitreous hemorrhage, approximately six disc diameters in size, involving the entire posterior pole (Figure 1). The LE showed no abnormalities. Based on the clinical history and examination findings, a presumptive diagnosis of VR was made. Considering the possibility of spontaneous resorption and the absence of peripheral retinal pathology, a conservative approach was initially adopted, with instructions for close follow-up and laboratory testing (complete blood count and coagulation profile).

Two weeks later, the patient returned reporting persistent visual blurring in the RE, with no improvement in BCVA (still hand motion). Laboratory tests showed no hematologic or coagulation abnormalities. He was referred for vitreoretinal surgical evaluation and ocular ultrasonography was ordered.

**Figure 2:**
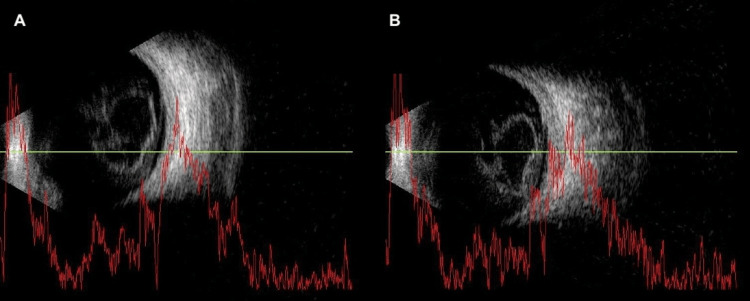
Ocular ultrasonography revealing hyperreflective lesion in the premacular space consistent with hemorrhage, associated with partial posterior vitreous detachment and discrete vitreous echoes, indicative of vitreous hemorrhage (A-B)

At five weeks after symptom onset, the vitreoretinal specialist re-evaluated the patient, who then reported mild subjective improvement. BCVA had improved to counting fingers at 0.5 meters in the RE. Fundoscopy revealed media opacity with limited visualization of the posterior pole, and a white, clumped lesion in the inferior nasal retina, likely consistent with hemorrhage. B-scan ultrasonography (Figure 2) confirmed a dome-shaped, hyperreflective lesion in the premacular space consistent with hemorrhage, associated with partial posterior vitreous detachment and discrete vitreous echoes, indicative of vitreous hemorrhage. Optical coherence tomography (OCT) was not performed due to media opacity. Given the lack of functional recovery and persistent hemorrhage, surgical intervention was indicated.

At eight weeks, the patient underwent 25-gauge PPV, with surgical access facilitated by adequate media clarity and lens transparency, thereby eliminating the need for concomitant phacoemulsification. Nd:YAG laser membranotomy was not performed due to a higher risk of macular complications and the unavailability of the specific contact lens required for the procedure in the facility. The surgical procedure was uneventful.

**Figure 3:**
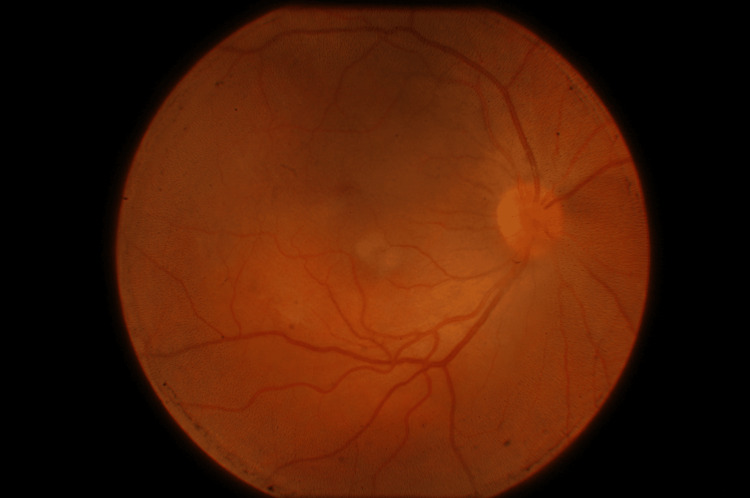
Retinography performed on the 10th postoperative day showed no residual hemorrhage or structural abnormalities

Postoperatively, the patient was prescribed moxifloxacin eye drops, prednisolone eye drops, tropicamide eye drops, and oral nonsteroidal anti-inflammatory drugs. On postoperative day 10, he reported significant visual improvement. Prednisolone was being tapered, and tropicamide was still in use. BCVA in both eyes was 20/20. Fundus photography showed no residual hemorrhage or structural abnormalities (Figure 3).

After completing the postoperative medication regimen and re-evaluation four weeks later, the patient was discharged from follow-up care.

## Discussion

VR is typically a benign, self-limiting retinal condition that arises from a sudden increase in intrathoracic or intra-abdominal pressure - most often during activities like weightlifting, vomiting, or coughing. This abrupt pressure surge disrupts fragile perifoveal capillaries, usually resulting in hemorrhage beneath the internal limiting membrane (ILM) or in the subhyaloid space, most often involving the macula [1-4]. For many patients, especially those with small to moderate hemorrhages, the condition resolves spontaneously, without lasting damage. However, when the hemorrhage is dense, extensive, or centrally located, this natural course may not unfold as expected.

In our case, the patient was a healthy young adult with no systemic illness, yet he developed a large premacular hemorrhage following a Valsalva maneuver during physical exertion. Conservative management was initially chosen, consistent with published guidelines recommending observation in the early weeks [2,5,6]. However, after eight weeks of follow-up, the patient’s visual acuity remained at hand motion, and clinical and ultrasonographic findings showed no sign of resolution. At that point, the prolonged blood contact with the macula raised concern, not only for the risk of continued visual impairment but also for potential toxic damage to the retina due to hemoglobin breakdown and oxidative stress, as previously described by Patane et al. and Li et al. [5,6].

The timing of intervention in VR is often debated. In our patient, the decision to proceed with surgical intervention at eight weeks was grounded in a combination of clinical and anatomical factors. He was a young individual with persistent visual loss and failure of conservative treatment. Ophthalmic examination and imaging revealed a firm vitreoretinal adhesion, which initially made early surgery less favorable. We deliberately allowed additional time in the hope of spontaneous posterior vitreous detachment and hyaloid layer impregnation, both of which would facilitate a safer and more controlled surgical approach. This patient-tailored strategy sought to optimize surgical conditions while minimizing intraoperative risks, particularly in a phakic eye. While some studies suggest a three-to-five-week window for observation, others emphasize individualizing decisions based on hemorrhage density, patient age, and functional impact [7,8].

Nd:YAG laser membranotomy is often considered a minimally invasive alternative, especially when the hemorrhage is strictly subhyaloid. However, its success depends on precise hemorrhage localization, media clarity, and the presence of PVD - all of which were lacking in our case. In addition, as Leite et al. and Brar et al. caution, laser treatment in patients without PVD may lead to complications such as macular hole, retinal traction, or persistent subretinal fluid [7,9]. For these reasons, and due to the lack of a suitable contact lens for safe laser delivery, Nd:YAG was deemed inappropriate.

Vitrectomy, while more invasive, offered the opportunity to clear the hemorrhage completely, minimize intraoperative risks, and achieve anatomical restoration. We opted for 25-gauge PPV, with successful induction of PVD and no need for ILM peeling, a strategy supported by Dulger et al., who advocate against peeling in young, phakic patients to avoid unnecessary retinal manipulation [10]. The surgical outcome was satisfactory: the patient recovered to 20/20 vision by the 10th postoperative day, with no complications observed throughout follow-up.

Imaging was central to our decision-making. B-scan ultrasonography and careful fundoscopic monitoring can provide critical insight into hemorrhage evolution, helping distinguish cases that may still improve spontaneously from those requiring intervention [11]. Other authors support early surgery when hemorrhage fails to resolve, noting not only the visual risks but also the psychological burden of prolonged visual disability in young patients [12].

This case reinforces the importance of individualizing care in VR. While observation is appropriate for many, clinicians should remain attentive to red flags: dense hemorrhage, persistent visual loss, absent signs of reabsorption, and no PVD. When these are present, particularly beyond four to six weeks, surgery becomes not just reasonable but likely necessary.

From a clinical standpoint, this case offers some key takeaways. Conservative management remains valid but requires active reassessment. Imaging should guide both diagnosis and timing of intervention. When surgery is indicated, minimally traumatic approaches like small-gauge PPV may restore vision safely and effectively.

## Conclusions

While many cases of VR resolve on their own, not all follow a benign course. In patients with dense, persistent hemorrhages and no signs of spontaneous improvement, timely surgical intervention can make all the difference. In our case, eight weeks of stable but severe visual impairment signaled the need to act. Vitrectomy not only cleared the hemorrhage but also fully restored the patient’s vision. This experience reinforces a practical message: when observation no longer serves the patient, clear clinical criteria - such as hemorrhage density, duration, and lack of posterior vitreous detachment - should guide the shift toward surgery. Recognizing the right moment to intervene is key to preserving vision and quality of life.
